# Acceptability of a pilot motivational interviewing intervention at public health facilities to improve the HIV treatment cascade among people who inject drugs in Indonesia

**DOI:** 10.1186/s12954-024-00989-w

**Published:** 2024-04-01

**Authors:** Lydia V. Wongso, Arie Rahadi, Evi Sukmaningrum, Miasari Handayani, Rudi Wisaksana

**Affiliations:** 1 University Center of Excellence - AIDS Research Center, Health Policy and Social Innovation, Atma Jaya Catholic University of Indonesia, DKI Jakarta, 12930 Indonesia; 2 Faculty of Psychology, Atma Jaya Catholic University of Indonesia, DKI Jakarta, 12930 Indonesia; 3 Research Center for Care and Control of Infectious Diseases, Universitas Padjadjaran, Bandung, 40161 Indonesia

**Keywords:** Motivational interviewing, Psychosocial intervention, Acceptability, HIV, People who inject drugs, Adherence, Antiretroviral therapy

## Abstract

**Background:**

HIV-positive people who inject drugs (PWID) experience challenges in initiating and adhering to antiretroviral treatment (ART). Counselling using motivational interviewing (MI) techniques may help them formulate individualised strategies, and execute actions to address these challenges collaboratively with their providers. We evaluated the acceptability of MI from a pilot implementation at three public health facilities in Indonesia.

**Methods:**

Adapting the acceptability constructs developed by Sekhon (2017) we assessed the acceptability to HIV-positive PWID clients (*n* = 12) and providers (*n* = 10) in four synthesised constructs: motivation (attributes that inspire engagement); cost consideration (sacrifices made to engage in MI); learned understanding (mechanism of action); and outcomes (ability to effect change with engagement). We included all providers and clients who completed ≥ 2 MI encounters. Qualitative analysis with an interpretive paradigm was used to extract and categorise themes by these constructs.

**Results:**

In motivation, clients valued the open communication style of MI, while providers appreciated its novelty in offering coherent structure with clear boundaries. In cost consideration, both groups faced a challenge in meeting MI encounters due to access or engagement in other health care areas. In learned understanding, clients understood that MI worked to identify problematic areas of life amenable to change to support long-term ART, with reconciliation in family life being the most targeted change. By contrast, providers preferred targeting tangible health outcomes to such behavioural proxies. In outcomes, clients were confident in their ability to develop behaviours to sustain ART uptakes, whereas providers doubted the outcome of MI on younger PWID or those with severe dependence.

**Conclusions:**

There is broad acceptability of MI in motivating engagement for both actors. Relative to providers, clients were more acceptable in its mechanism and had greater confidence to perform behaviours conducive to ART engagement. Design innovations to improve the acceptability of MI for both actors are needed.

**Supplementary Information:**

The online version contains supplementary material available at 10.1186/s12954-024-00989-w.

## Introduction

Despite the universal eligibility of adult antiretroviral treatment (ART) in Indonesia, disparities in the HIV care cascade by HIV key population status and particularly among people who inject drugs (PWID) persist [1]. Findings from a multicentre cohort show that of all HIV key populations, PWID experience the largest drop in the cascade of care following HIV diagnosis and a higher prospect of treatment failure despite having similar rates of ART retention [2]. In the wake of these findings, the HIV *Awal* (early) Testing and Treatment Indonesia (HATI) study developed and piloted a counselling with motivational interviewing (MI) approach as an intervention targeting ART initiation and reinforcement of adherence behaviours during early ART among HIV-positive PWID. The current paper reports on the acceptability of MI to PWID clients and providers from the project.

MI originated in the field of substance abuse to motivate drug-use reduction [3] and has seen successful applications in management of chronic conditions requiring major changes in health behaviours to accommodate and comply with the prescribed long-term treatment regimens [4, 5]. MI is a collaborative, person-centred conversation to articulate and resolve ambivalence regarding a behaviour change by exploring the patient's intrinsic capacity in motivating actions towards a set of personalised objectives favouring change [6, 7]. Mounting evidence underscores fidelity to the principles of MI collaborative communication (i.e., using open-ended questions, affirmation, reflective listening, and providing summary reflections) [4, 7], with demonstrable effects being attainable in no more than eight weeks and possible with brief encounters [5, 8]. These features have led to MI adoption in various resource-constrained settings where the existing pool of medical professionals are expected to accommodate MI delivery with tailored training [9–11].

Although HIV is the chronic disease most extensively studied to ground evidence for MI effectiveness in promoting treatment adherence, findings relevant to specific comorbidities prevalent in people living with HIV such as injecting drug use remain scarce. The few existing efficacy reports in people with alcohol or crack cocaine problems have produced mixed results [12–15]. Moreover, a growing number of reports on increasing trends in attrition during ART suggest that universal ART eligibility may attract more patients with low motivation to adhere. They would have likely dropped out of pre-ART care had eligibility been determined on the basis of immunological/clinical criteria [16–18]. Therefore, introducing MI following HIV diagnosis would be a promising strategy to motivate ART initiation and encourage adherence behaviours in PWID during early ART—the critical period in which the risk of mortality and treatment discontinuation peaks [19]. In addition to the current focus of MI assessments in reversing poor adherence, its efficacy in facilitating ART initiation has been demonstrated when packaged with other support and psychosocial interventions [20].

The foregoing sets the background for our pilot implementation of MI in Indonesia. The current paper contributes to the evidence base for feasible implementation of this approach and provides such an assessment in a practice setting. Therefore, our objective is to evaluate the acceptability of MI in promoting and retaining HIV-positive PWID in ART care, from the perspectives of clients and providers.

## Methods

### Overview of HATI study

This study was embedded in the second phase of HATI Project aimed to assess the feasibility and effectiveness of add-on interventions at selected HIV outpatient clinics to promote rapid initiation and retention of ART during the 2018–2019 period in four districts. MI as one of the add-on interventions being piloted was evaluated in a single-arm, non-randomised design at three sites: two health centres in the capital city Jakarta, and one provincial referral hospital in Bandung, the capital city of West Java. All sites are state-owned health facilities staffed by government employees. At the time of the study, both health centre HIV clinics were staffed with two clinical professionals and one general staff member, serving an average load of 200–300 ART patients per month. The hospital HIV clinic, on the other hands, was managed by six clinical professionals and six general staff members, with an average load of 2,000 ART patients per month.

HATI also piloted the following two other add-on interventions: (a) Simplified ART Initiation protocols, with supporting laboratory assessments scheduled within two weeks of initiation as opposed to being a prior requisite for initiation in the current practice; and (b) Appointment reminder for clinical follow-up and antiretroviral refills through short message service (SMS) on mobile phones. The first phase of HATI Project was an observational cohort evaluating the cascade of HIV care beginning immediately after HIV diagnosis [2]. An effectiveness evaluation of the MI pilot has been reported elsewhere [21]. Details on the design and the intervention mix piloted in each of the four intervention districts are available at clinicaltrials.gov (NCT03659253).

### MI intervention procedures

Preparatory work was conducted from February to December 2017 to develop MI modules [22], train providers, set up venues, and trial MI under direct supervision on three to five eligible clients per clinic to familiarise providers with MI procedures and fine-tune modules as appropriate. MI providers were existing health care staff at respective clinics with qualifications in HIV and addiction counselling. All clinics began MI enrolment in January 2018. Fidelity and other aspects of quality assurance were upheld by a team of psychologists and health researchers who held weekly case conferences with the providers and conducted monthly site visits throughout the intervention period. Patients aged ≥ 16 years who presented with an HIV diagnosis, were either ART-naive or had discontinued ART in the past ≥ 3 months, and reported history of injecting drug use were offered MI during routine medical visits. Consenting patients underwent brief examinations of depression-anxiety and stress (DASS-21) [23], adequacy of social support network (SSNQ) [24], and additionally, medication adherence self-efficacy (HIV-ASES) [25] for those who were initiating ART. The results of these examinations determined the current stage of change of MI clients and guided the selection of appropriate counselling modules to match in the subsequent MI encounter (see Additional file 1). In MI, change is presumed to occur in progressive stages describe the readiness of a client for change and counsellors tailor their approach around this stage to inspire motivation to change [22]. All subsequent MI encounters were scheduled within 30 days of the last attended session. Clients who missed their MI appointments were contacted to reschedule within a month. MI clients completed participation either after attending an allotted 10 MI encounters or upon reaching the terminal stage of change ('maintenance') within 12 months post-enrolment.

### Qualitative study design

#### Acceptability constructs

We define acceptability as the extent to which providers and clients considered MI to be appropriate in motivating ART uptakes and adherence. Operationally, we used the Theoretical Framework of Acceptability (TFA) developed by Sekhon and colleagues [26] to guide our design of semi-structured interview questions because of its explicitness and its range of multifaceted constructs in: (a) *affective attitude* (how an individual feels about MI); (b) *burden* (the effort required in delivering or obtaining MI); (c) *ethicality* (agreement with personal values); (d) *intervention coherence* (how participants understand the intervention logic of MI); (e) *opportunity costs* (activities and resources forgone to participate in MI); (f) *perceived effectiveness* (the perceived likelihood of MI in promoting ART uptakes and adherence); and (g) *self-efficacy* (how confident the necessary behaviours required to participate in MI can be performed). Sample interview questions include “What did you feel initially about the MI?” under the *affective attitude* construct and “How, if there was any, did you trade-off a portion of your daily routines to commit to MI sessions and solutions?” under the *opportunity cost* construct. Probing questions that followed distinguished responses by groups of study participants as MI providers or clients and were framed to explore possible changes in attitude and perceptions towards MI as over time providers and clients accumulated experience from more MI encounters.

#### Participants, recruitment, and data collection

We recruited and interviewed MI participants in two groups. The provider group comprised 10 health care staff who were trained and assigned to deliver MI counselling. The client group comprised PWID patients who completed at minimum two MI encounters by October 2018 and was purposively sampled in consultation with the providers who recommended candidate participants who had completed the intervention (*n* = 4) and those who remained on-going with the intervention (*n* = 8).

Participants were interviewed in a private room by a research team who had no affiliation with the study sites. Each interview opened with a brief background of MI to establish a reference to its theorised intervention logic, which participants were expected to evaluate in terms of its applicability and appropriateness by each TFA construct described above. Duration of interviews ranged from 25 to 90 min. All interviews were audio recorded.

### Data analysis

Transcripts from audio recording were deductively coded according to the TFA constructs for thematic analyses [27]. We first identified initial qualitative themes across the seven TFA constructs, which we refer to as sub-themes. by participant group. We used an inductive process in the next stage of analysis in which we examined the degree of overlap in meanings across these sub-themes to synthesise the initial constructs into four final constructs describing *motivation* (*affective attitude* and *ethicality*); *evaluation of costs and benefits* (*burden* and *opportunity cost*); *learned understanding of MI* (*intervention coherence*); and *MI outcomes* (*self-efficacy* and *perceived effectiveness*). LVW and ES conducted all coding and analyses independently with a third author adjudicating (AR) disagreements in thematic classifications and labelling of sub-themes. We conducted a group validation meeting in which provider participants were asked to confirm their responses and comment on the preliminary results relevant to the provider group. Due to difficulties in scheduling a group meeting for client participants, no validation meeting was conducted for this participant group. NVivo version 11 was used to analyse qualitative data (QSR International Pty Ltd, 2015).

### Ethics statement

All participants provided written informed consent and received small gifts and transport reimbursements equivalent to USD8.00 in value for their participation. The study was approved by the Committee of Research Ethics of Atma Jaya Catholic University of Indonesia (ref: 0025/III/LPPM-PM.10.05/01/2019).

## Results

### Informant characteristics

Our informants comprised 12 clients and 10 providers (Table 1). The client participants were predominantly male, averaged 35 years in age, with a greater number recruited in Jakarta, having current employment, having completed secondary education, and having initiated ART at the time of interview. The provider participants averaged 38 years of age with a broader range of distribution compared to the clients, were overwhelmingly female, and had an equal split between those who were medical professionals (i.e., medical doctors and registered nurses) and those who were not (i.e., psychologists and counsellors).

**Table 1:** Characteristics of participants

Characteristic	Participant group (n)	
	Clients	Providers
n participants	12	10
District		
Bandung	4	4
Jakarta	8	6
Health facilities		
Hospital	4	3
Health centre	8	7
Age in years, mean (range)	35 (30–42)	38 (24–64)
Sex		
Male	11	3
Female	1	7
Education		
Less than high school	1	–
High school	7	–
University/academy	4	–
Employment status		
Employed	8	–
Unemployed	4	–
Marital status		
Never married	4	–
Currently married	5	–
Divorced/widowed	3	–
ART status		
On ART	10	–
Not on ART	2	–
MI status		
Completed	4	–
On-going	8	–
Health care profession		
Medical doctor	–	3
Nurse	–	2
Psychologist/counsellor	–	5

*ART* Antiretroviral treatment, *MI* Motivational interviewing

### Summary of sub-themes by TFA construct

We identified a total of 16 and 17 sub-themes for the client and provider groups, respectively, by the TFA construct (Table 2). In *affective attitude* clients emphasised sub-themes related to freedom in communication (Ref. code A-FC-01 in Table 2) despite some uncertainty regarding how this “talk therapy” would work to motivate uptakes and adherence of ART (A-BC-02). A similar sub-theme of freedom in communication was reiterated in *ethicality* (B-FC-01). Next, *burden* and *opportunity cost* correspond to challenges in life (C-BC-01) and daily activities sacrificed to commit to the MI process (D-BC-01) and collectively represent a cost consideration in deciding attendance to MI sessions. In *intervention coherence* clients expressed how MI helped them acknowledge the strained relations in their close social network due to past drug-use behaviour as a major barrier to social support (E-FC-01). Clients generally felt confident in their *self-efficacy* to commit to MI encounters (F-FC-01) and gradually consolidate social support which they viewed as a key success indicator of *perceived effectiveness* (G-FC-01, G-FC-02) as they cycled through the stages of change.

**Table 2:** Sub-themes and TFA constructs by participant group

Acceptability construct (*definition*)	Client		Provider	
	Ref. code	Sub-theme	Ref. code	Sub-theme
A. Affective attitude (*How an individual feel about MI*)	A-FC-01	A medium to express views freely and be listened to*	A-FP-01	A novel behavioural therapy with a prospect of a new skills acquisition*
	A-FC-02	Mutual understanding and support in deciding to initiate HIV treatment*	A-FP-02	MI empowered and facilitated to explore clients’ problems*
	A-BC-01^†^	Inconsistent engagement leading to discomfort^†^	A-BP-01	Extra time and effort in the learning curve to effectively deliver MI^†^
	A-BC-02	Initial doubts over MI as another form of “talk therapy”^†^	A-BP-02	Limited capacity of MI to engage clients with severe drug use^†^
B. Ethicality (*The extent to which MI has good fit with an individual's value system*)	B-FC-01	Freedom to express emotions and articulate ideas were cathartic and encouraged truthful conversations*	B-FP-01	Re-affirmation of a client's problems through active listening acknowledged their dignity and appreciated their participation*
			B-BP-01	Provider-centric values with emphasis on abstinence, compliant ART, and other noble health pursuits hindered MI adaptation*
C. Burden (*Amount of effort that is required to participate in MI*)	C-BC-01	Unique life circumstances posed a chall- enge to participation in fixed schedules^†^	C-BP-01	Other clinical and clerical duties posed a challenge to sustained MI delivery^†^
	C-BC-02	Discomfort in detaching oneself from deep-seated problems for planning an effective course of action^†^	C-BP-02	“Trials and errors” in reconciling MI principles with preferred counselling style^†^
D. Opportunity cost (*The extent to which benefits, profits or values must be given up to engage in MI*)	D-BC-01	Disruptions in daily routines due to MI sessions being scheduled at the provider’s convenience^†^	D-BP-01	Forgoing other duties to accommodate extra time for MI^†^
E. Intervention coherence (*The extent to which the participant understands MI and how it works*)	E-FC-01	MI exposed underlying social relations and unmet expectations that deviated clients from their care*	E-FP-01	MI built on the client's personal achievements towards a health objective*
			E-FP-02	MI demands careful attention to the client's needs and their acknowledgement and contributions in resolving a health problem*
	E-FC-02	Solutions in MI are reflective of subjective life circumstances as opposed to be-ing prescriptive		
	E-BC-01	An unmet expectation of a ‘closure’ or 'milestone' after each session^†^		
F. Self-efficacy (*The participant's confidence that they can perform the behavior[s] required to participate in in MI*)	F-FC-01	High confidence in adhering to MI sessions and negotiating among the daily activities to commit to change*	F-BP-01	Generational gaps in the client-provider relationship posed a challenge^†^
			F-BP-02	Confusion in determining the appropriate stage of change^†^
	F-BC-01	Doubts if the resulting change was durable^†^	F-BP-03	Compromised commitment to MI due to competing duties^†^
G. Perceived effectiveness (*The extent to which MI is perceived as likely to achieve its purpose*)	G-FC-01	MI confronted long held irrational beliefs and situated the problem around the interactions with personal and professional aspects of life*	G-FP-01	MI helps clients deal with social problems driving medication non-adherence*
			G-FP-02	HIV treatment adherence is the first step towards improvements in quality of life*
	G-FC-02	MI worked to resolve social conflicts in order to expand support resources and enable change*	G-BP-01	Effectiveness is contingent on the severity of client’s drug use^†^
	G-BC-01	Prioritising which conflicts to resolve can be a challenge^†^		

*ART* Antiretroviral treatment, *MI* Motivational interviewing, *TFA* Theoretical framework of acceptability

*Facilitators

^†^Barriers

For providers, MI was approached with a sense of novelty and enthusiasm in *affective attitude* (A-FP-01). In *ethicality* they acknowledged a challenge in transitioning to the client-centred principle of MI from the established paternalistic practice in health behaviour counselling (B-BP-01). Providers discussed challenges in balancing commitment to MI appointments and other clinical or clerical duties, and perceived the pilot MI as being disruptive to their established professional roles in *burden* (C-BP-01) or *opportunity cost* (D-BP-01). In *intervention coherence*, providers characterised the stages of change in MI as having incremental target actions (E-FP-01) adaptable to the unique life circumstances of the clients (E-FP-02). In *self-efficacy* providers felt being less than optimal in MI delivery, which they attributed among others to the gap in skills to communicate to younger clients in the pilot period (F-BP-01). Finally, providers associated *perceived effectivene*ss with how invested clients were to gradually build a foundation of support for long-term ART (G-FP-01, G-FP-02).

### Themes in final acceptability constructs by participant group

**Fig. 1:**
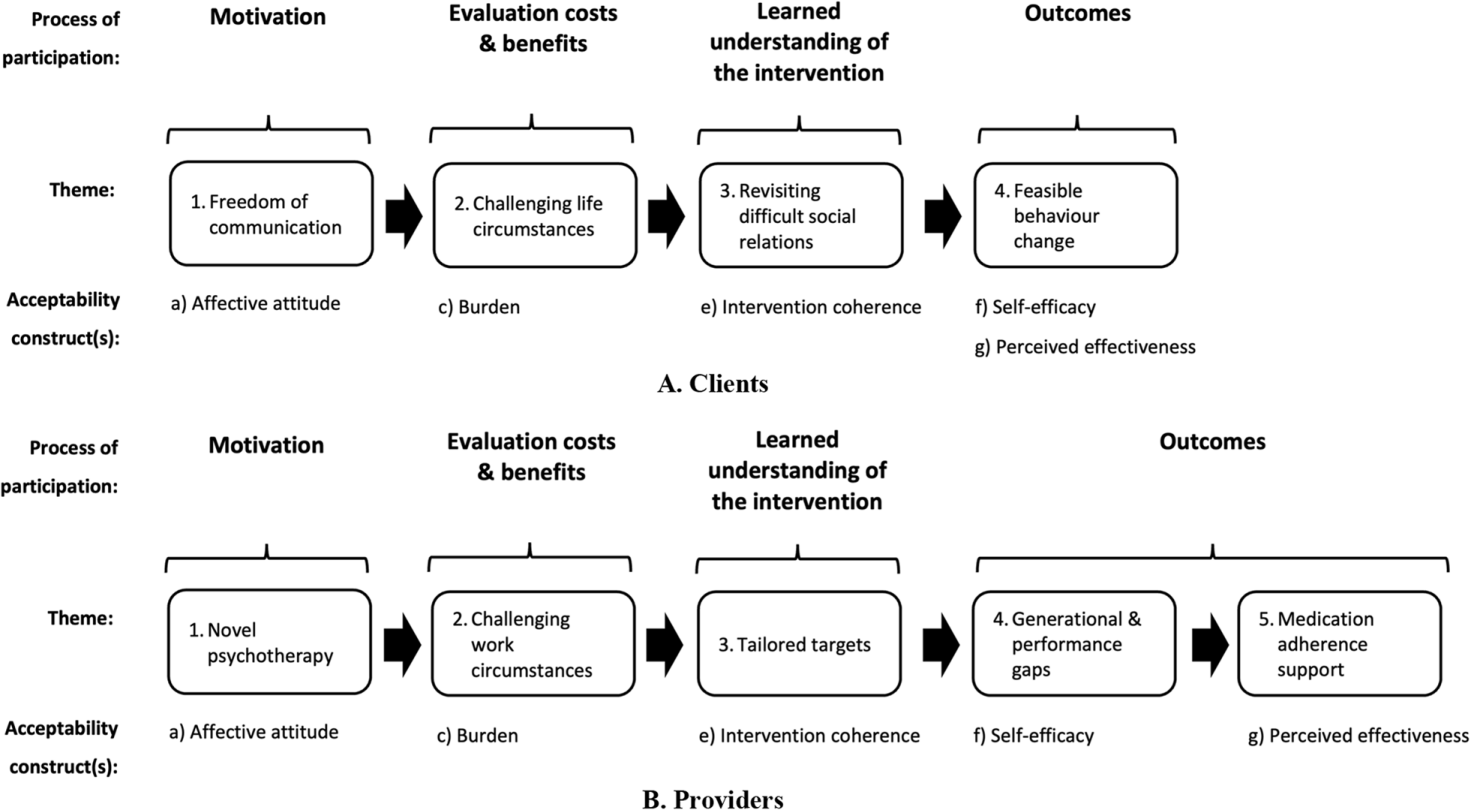
Final acceptability constructs and themes by participant group. The figure presents study qualitative themes organised by groups of theoretical framework of acceptability comprising seven constructs synthesised by Sekhon et al. [26]. ‘Affective attitude’ pertains to how an individual feels about MI. ‘Burden’ captures the amount of effort to participate in and complete MI sessions. ‘Opportunity cost’ measures benefits sacrificed or activities forgone to participate in and complete MI sessions. ‘Intervention coherence’ reflects how participants understand the intervention logic of MI. ‘Self-efficacy’ refers to confidence to perform standard behaviors required in MI participation. ‘Perceived effectiveness’ evaluates how MI is perceived as likely to achieve its purpose. Prominent acceptability constructs and their corresponding themes are highighted along the stages of participation. *MI* Motivational interviewing, *TFA* Theoretical framework of acceptability

A description of qualitative themes formulated from synthesising relevant sub-themes and constructs by participant group is provided in Fig. 1. Themes are ordered along a continuum of process of participation in MI, beginning with the *motivation* to engage in MI and terminating with *MI outcomes*.

### MI client group

#### Motivation

Clients perceived *freedom of communication* as an important attribute of MI during motivated encounters and participation. Clients reported an initial struggle in adapting to the open communication style of MI, which was perceived to offer minimal directions to draw attention to important aspects of life that may have a bearing on treatment performance. However, once clients had successfully narrowed down these aspects to focus areas, they felt that MI discussions encouraged self-awareness of current beliefs and inventorying probable enablers and barriers to ART uptake. This process gave clients a sense of hope in adapting to MI and transitioning the therapeutic engagement to a more active role over the course of the intervention:

“As I'd brought myself to complete the first few sessions, I began to feel good and happy. I felt a great sense of relief as I could offload some of the worries I had harboured [regarding ART] and embrace them for what they are.”—Client A, Jakarta.

#### Evaluation of costs and benefits

Commitment to participate in the intervention was met with negotiating and, at times, sacrificing daily activities. Current engagement in informal/unstable work and caring duties in the household presented *challenging life circumstances* in adhering to scheduled MI encounters in addition to other health care visits (e.g., ART, drug substitution treatment), which could lead to lost earnings or extra expenses to find a substitute career. Additionally, clients believed that frequent absenteeism from work or other regular activities would lead to unintentional disclosure of their HIV status and result in discrimination. In response, clients resorted to rescheduling MI encounters to prioritise immediate needs and maintain good social standing in their household:

*“The only catch is when they set the schedule during work hours – I need to be at work early and then go to the clinic and return to work after I am done with my health care business… Let alone [if there is] an urgent need at home I need to attend to. So, what am I to do?” —Client B Jakarta.*

#### Learned understanding of MI

The open communication principle of MI allowed clients to reflect on their experiences and *revisit difficult social relations* in their immediate social network as a consequence of their habitual drug use. In the process they expressed that they developed a gradual need for tackling unresolved conflicts with their loved ones as they became increasingly aware of their own faulty beliefs. The importance of trust rebuilding appeared motivated to prepare disclosure of their HIV status with an expectation to be reciprocated by gains in emotional or other support during chronic ART. As one client recounted:

*“I remember after I just finished serving my time [in prison] my mother would go ballistic on me, saying '*You are good for nothing and can’t even provide for your kids*!'… After all she is the only one in the family who knows my HIV status. So, I enrolled in MI and started my counselling. Wow, she [the provider] really digs into my head… to the extent I think she could feel what I felt. I could relate to much of what she said and finally tried to leave my comfort zone. It took me a while, but with some effort eventually I could find work. So, one day I shared the news with my mother. I also showed her my viral load test results which are undetectable and told her what little money I saved from work to provide for my kids and in-laws. She responded, 'Wow, you’ve changed!' I cried when I heard her say that.”—Client B Jakarta.*

#### MI outcomes

Clients attributed MI to helping them in finding sensible solutions that were sufficiently contextualised to the problems identified in their self-reflection, thereby prompting *feasible behaviour change*. While clients regarded the overall effort to participate in MI as being acceptable, confidence in execution may be impacted for those in the later stages of change as routine ART visits became embedded in their daily lives and added to the existing health care visits. In judging the perceived effectiveness of MI, clients pointed out that the personalised pace of counselling afforded them time to reflect on their current beliefs, confront them, and re-build a support network with the individuals whose support they regarded as essential:

*“I struggled in the first session and tried so hard to find the goals I think mattered to me. Towards the end of the session, we began to talk more seriously about my poor habit of not consistently sticking to my medication time—I used to think missing taking my medication by one or two hours would still do me as good. But that was the problem and I was not aware there would be consequences of my poor habit because I didn’t feel anything, yet. So, I set my goal to be consistent with my medication time at the end of the first session. I made sure I got all the support I needed, including getting a peer support service to remind me of my medication time. Then came the second session, and after that I felt more comfortable to dot all the I’s and cross all the T’s when it comes to my medication. I felt proud of myself!”—Client A, Bandung.*

### MI provider group

#### Motivation

Providers approached MI as a *novel psychotherapy* that presented an opportunity for professional growth by mastery of new skills and also brought in additional duties in health care delivery. Providers with substantial experience in drug treatment programs viewed MI as a promising motivational intervention to effectively manage drug use, which would facilitate ART initiation and adherent uptakes. Although paradoxically providers voiced a concern that the client-centred counselling dynamics in MI may lack directives to prioritise reduction or cessation of drug use, a health message they strongly upheld in the traditional drug use counselling, there was an agreement that the clarity of structure in MI brought coherence to what was expected with each session:

*“With the traditional counselling there was a total absence of guidelines or directions in what we were supposed to do and how we were supposed to do it. Anything goes, which oftentimes led the counselling to be off-focus and all over the place. With MI we are given a clear set of boundaries. And whenever we felt we no longer maintained the momentum [in a counselling session], it was easier for us to regain focus by being cognisant of those boundaries.”—Counsellor 1, Jakarta.*

#### Evaluation of costs and benefits

The way MI was systematically structured underlined the need to accommodate continuous encounters in contrast to the traditional counselling whereby individual sessions were allocated on-demand at the request of clients. In this view MI was perceived to add to the existing provider duties in both clinical and clerical work, with a possible challenge in committing to scheduled MI sessions. Providers recounted their struggle with *challenging work circumstances* in navigating through competing duties in various health programs, prompting rescheduling and a feeling of ambivalence in conducting MI sessions, with implications for MI fidelity and effectiveness:

*“We [staff at Health Centres] must divide our time among various tasks, especially for staff like me. Health Centre management comprises individual care, often quite clinical, and the promotive care for the masses, which is quite educational. I stand in both roles and have to carefully balance between these two broad programs. Since our Health Centre gained national accreditation, there has been an increasing pressure to perform well in both areas programmatically and administratively. If I was too involved in promotive care, I would pass my clinical duties to someone else—and that is how things work here. We divide and delegate duties among staff whenever necessary.”—Counsellor 2, Jakarta.*

#### Learned understa﻿nding of MI

Providers understood MI as a continuous process that builds incrementally on *tailored targets*, demanding an appreciation of the unique individual circumstances of the clients. As one provider recounts:

*“If we had an understanding of MI techniques, this then could give us a sense of direction in how we should proceed in the counselling process… [and] where the patient was with regards to his/her stage of change. Typically, we can come up with creative ways to move clients through the stages of change if we’ve understood enough what MI is about. This would help us find unique solutions for unique problems and experiences that clients may have.”—Counsellor 3, Jakarta.*

When quizzed on the extent these personalised targets contributed to the public health objective of ART adherence, there was an agreement that these were critical, observable indicators of behaviour change to monitor progress. However, concerns were voiced on whether these personalised behavioural targets would provide valid proxies of future ART adherence.

#### MI outcome﻿s

There are two related themes in this merged construct. First, providers felt increasingly aware of a threat of *generational and performance gaps* in communicating the vital importance of ART adherence to younger clients who they perceived to have not matured in healthy lifestyle and experience with health care interactions.

*“Maybe because we are different in that the client was of young age and the way he catches on and processes things that are happening around him may differ from how I do on an intellectual level [as an older person]. These are all the obstacles I well acknowledge to come from me internally.”—Counsellor 4, Bandung.*

Second, the prospect of MI being efficacious was contingent on how effective clients were at building *medication adherence support*. In this view, providers believed that essential support that clients may have secured from the family and HIV-related services could be compromised by drug use severity, in favour of those who had less severe drug use. Recommendations were made for implementing an active referral system to drug treatment services or integrating cognitive behavioural therapy in MI delivery, particularly for cases involving severe drug use.

*“In my opinion MI cannot be expected to single-handedly tackle all the problems associated with drug use. It may well be the case that a combination approach with other counselling approaches such as cognitive behavioural therapy is called for.”*—*Counsellor 3, Jakarta.*

## Discussion

Our results suggest broad acceptability of MI among HIV-positive PWID clients as regards the motivation to engage in MI, learned understanding of its engagement, and its self-efficacy outcomes. From the perspective of providers, acceptability was most pronounced in the motivation domain. The coherent structure, principles of client-centred communication, and expectations embodied in MI were the main motivation for engagement by the providers and were perceived as a valuable competency worth acquiring to support their professional career. Moreover, providers perceived the structured flow and clear boundaries of MI as guiding the process of behaviour change for clients in piecemeal, more manageable steps in contrast with traditional counselling methodology. A previous study found that implementing a client-centred approach in family planning services enhanced client-provider communication dynamics, contributing to increased client satisfaction [28, 29]. We believe that a similar mechanism was replicated in our pilot in that the clear boundaries that defined MI-consistent behaviours helped keep the collaborative dynamics in check throughout the process of personalised goal setting and its attainment, leading to satisfaction among clients.

Juxtaposing the perspectives on what clients and providers found acceptable in MI, the findings suggest two target areas of realignment for future implementation. The first area of realignment pertains to the systematized structure of MI delivery exacting commitments from both clients and providers to scheduled encounters, and this is evident when evaluating the costs and benefits of participation. Not only would clients need to budget for extra health care visits for MI in addition to routine management of their comorbidities, but these extra visits also posed a risk of loss of earnings or a threat of mistrust over falling short of their caring duties in the household. Given the supply-side constraints in health care workforce [30], the introduction of stand-alone MI programming such as our pilot would disrupt the existing routine service delivery at public health facilities for which ad hoc solutions by delegating MI to staff with little or no training in MI may compromise the overall effectiveness of the intervention.

Reducing and fine-tuning the frequency of MI encounters that at minimum may range from one to three encounters may overcome this challenge [31]. An alternative to stand-alone programming is to integrate MI techniques with other motivational enhancement or cognitive behavioural therapies adapted to flexible modalities (e.g., during community outreach, clinic visits) as has been demonstrated in a successful randomised controlled trial [32]. Packaging MI as such can distribute the burden in delivery across multiple health care cadres and apportion motivational methodology appropriately as the needs arise. The matching motivational methodology or interventions to complement MI should be carefully selected to avoid MI-inconsistent behaviours such as directing [33], which can have deleterious effects [34].

The second area of realignment relates to the contrast in confidence between clients and providers in MI to target the expected behaviour change. While clients appeared to accept the notion of gradual transitions in behaviour change in MI more readily, providers did perceive a gap in whether and how MI can advance these transitions towards health outcomes despite their understanding of its gradual process. Ample instances of these transitions revolve around attempts at reconciliation in family to secure support [35], a process that can often be protracted and may impact providers' confidence in the ability of MI to elicit health outcomes more meaningful to HIV programming such as ART retention. Our longitudinal assessment of the pilot MI indicates that retention was similar between MI participants and non-participants [21], highlighting the need to optimise MI in the direction to sustain outcomes.

Other aspects of confidence in MI pertains to a perceived client-provider age gap and differential outcomes based on drug-use severity. The age gap in some client-provider relationships may reveal suboptimal planning of MI delivery, leading to a mismatch in age or other pertinent attributes in a counsellor/provider that clients may consider important in therapeutic relationships [36]. The overlapping existing duties of MI personnel may have contributed to the allocation of personnel on the basis of convenience rather than the needs of clients. Routine case conferencing can be useful to resolve this and many other issues in coordinating MI delivery among the providers. With regards to drug-use severity, putting in place a referral service for clients seeking drug treatment for their severe drug use can help prioritise MI provision to clients who are better prepared to manage their drug use and [37], consequently, adhere to lifelong ART [38].

One limitation of our study is that we included a limited number of participants in both groups. While the pool of providers, of whom we interviewed all, was fixed at this number, we were unable to recruit more eligible clients within the planned data collection period. The consequence is that the qualitative information in some acceptability constructs might not have reached saturation. Secondly, our sample of clients were identified and referred by the providers with whom they had routine service encounters for MI or other care. Therefore, social desirability was possible. Third, we did not assess the fidelity of MI during practice with an analysis of compliance to MI and client motivation revealed in the patterns of “change” or “sustain” talk [6]. Adapting to MI may have presented a challenging transition for providers who were accustomed to directive counselling aimed at advancing certain health objectives (e.g., abstinence from drug use), and possible deviations from MI as well as the extent to which they would negate outcomes were not captured in this study. Finally, our findings cannot be generalised beyond the participating sites; and neither did these sites, purposively selected in our pilot because of their resource endowments, represent a typical HIV care facility in urban Indonesia. Despite these limitations our study included perspectives of both clients and providers and provide important insights for programming MI or psychosocial interventions for HIV-positive PWID.

Considering the promising findings from the pilot on increased ART initiation [21], integrating MI techniques in community-based work with a focus on behaviour change communication can extend the benefits to clients in non-clinical settings. This approach is particularly helpful to reach PWID who may have barriers to health facility-based encounters. Future research endeavours should be directed to explore alternative MI modalities appropriate for the HIV-positive PWID population and ways to adapt MI principles in routine health care encounters and to the existing workforce skill-mix. Assessments of factors contributing to successful MI engagement and fidelity in relation to health outcomes, along with the moderating influence of drug use severity, should also be pursued, utilising a sufficiently large sample.

## Conclusion

The pilot MI implementation was acceptable in motivating HIV-positive PWID clients and providers to engage in the intervention, offering a novel, alternative form of counselling practice compared to the existing methodology. The understanding that MI examines various aspects of life that are modifiable was not matched by the same enthusiasm among the providers who prioritised tangible health outcomes as a direct consequence of sustained ART uptakes. However, both groups made considerable trade-offs to meet the demand of additional health service encounters from MI, thereby exerting an extra burden to access and provide the service. Designing a more efficient modality is warranted to improve the overall acceptability of MI to clients and providers.

### Supplementary Information

**Additional file 1.** MI process and session modules by stage of engagement.

## Data Availability

De-identified data may be available from the team on reasonable request.

## Abbreviations

ART: Antiretroviral therapy
DASS-21: Depression anxiety stress scales-21 items
HIV-ASES: HIV treatment adherence self-efficacy scale
MI: Motivational interviewing
PWID: People who inject drugs
SSNQ: Social Support Network Questionnaire
TFA: Theoretical framework of acceptability
