# Supplementary material for: Acceptability of a pilot motivational interviewing intervention at public health facilities to improve the HIV treatment cascade among people who inject drugs in Indonesia
Source: Harm Reduct J. 2024 Apr 1;21:73. doi: 10.1186/s12954-024-00989-w (PMC10985935; doi:10.1186/s12954-024-00989-w)
Supplement: Supplementary file 1 — Additional file 1. MI process and session modules by stage of engagement. [file 12954_2024_989_MOESM1_ESM.docx]

**Supplementary 1.** MI process and session modules by stage of engagement

|  |  | **Assessment test** | **Passing criteria** | **Module to select** | **List of modules**  Pre-contemplation, Contemplation, or Preparation  Module 1 : Stages of change  Module 2 : Awareness of HIV risk and treatment  Module 3 : Awareness of psychological impact of  HIV infection  Module 4 : Harm reduction and drug use  Module 5 : Building hope  Module 6 : Expressing fear  Module 7 : Personal values  Module 8 : Pros and cons of ART  Module 9 : Social relations  Module 10 : Individual roles in society  Module 11 : Self-Confidence and Temptation  Module 12 : Problem-solving skills  Module 13 : Set Final Goal and Preparation to Change  Module 14 : Evaluation of MI sessions  Action or Maintenance  Module 15 : Stages of change in 'Action' and 'Maintenance'  Module 16 : Identifying triggers of non-adherence  Module 17 : Making an action plan  Module 18 : Reconnection to care following treat- ment interruptions  Module 19 : Social support  Module 20 : Identifying needs and resources  Module 21 : Review and termination |
| --- | --- | --- | --- | --- | --- |
|  |  |  |  | **if criteria not** |  |
|  |  |  |  | **met** |  |
| **Stage of MI engagement** | Preliminary | a) DASS inventory | All subtests scored 'no problem' | Modules 9-10 |  |
|  |  | b) SSNQ | >1 social contact in >4 domains of social support | Modules 9-10 |  |
|  |  | c) HIV/ART knowledge | Total score >12 | Module 2 |  |
|  |  | *Graduate to Evaluation of Barriers if a, b, and c passed* | |  |  |
|  | Evaluation of Barriers | a) Self-report adherence | Missed a <1 dose in past 30 days | Modules 3-5 |  |
|  |  | b) HIV-ASES | Average score >5 | Modules 5, 7-8 |  |
|  |  | *Graduate to Maintaining Adherence if a and b passed* | |  |  |
|  | Maintaining Adherence | a) Self-report adherence | Missed a <1 dose in past 30 days | Modules 12, 15 |  |
|  |  | b) HIV-ASES | Average score >5 | Modules, 11, 13 |  |
|  |  | c) Pill count | *#* prescribed = *#* consumed | Modules 12, 15 |  |
|  |  | *Graduate to Review & Termination if a, b, and c passed AND Modules 14, 16-17 completed* | |  |  |
|  | Review & termination | Modules 18-21 | | |  |
| ART: Antiretroviral treatment; DASS: Depression Anxiety Stress Scales; HIV-ASES: HIV Treatment Adherence Self-Efficacy Scale; MI: Motivational interviewing. | | | | | |
| All clients were assessed at each session using relevant tests for a stage of MI engagement, except for 'Review & Termination' in which clients were expected to complete the corresponding modules. The resulting test scores determined subsequent MI modules to deliver, progression to the next stage of MI engagement, or regression to the previous stage as deemed necessary by the providers. All participants received Module 1 on the stages of change in MI in the first session. The panel on the right lists the MI modules used in the pilot. | | | | | |
